# Supplementary material for: Dissociable neural correlates of uncertainty underlie different exploration strategies
Source: Nat Commun. 2020 May 12;11:2371. doi: 10.1038/s41467-020-15766-z (PMC7217879; doi:10.1038/s41467-020-15766-z)
Supplement: Supplementary file 1 — Supplementary Information [file 41467_2020_15766_MOESM1_ESM.pdf]

Dissociable Neural Correlates of Uncertainty Underlie Different Exploration Strategies: Supplementary Information

Momchil S. Tomov[1,2] , Van Q. Truong[2] , Rohan A. Hundia[2], Samuel J. Gershman[2]

[1]*Program in Neuroscience, Harvard Medical School, Boston, MA 02115, USA*
[2]*Department of Psychology and Center for Brain Science, Harvard University, Cambridge, MA 02138, USA*

Supplementary Information

Variance inflation factors

Since the parametric modulators of the trial_onset regressor are correlated (e.g. V and V/TU), we computed variance inflation factors (VIFs) for all four parametric modulators on all runs for all subjects (Supplementary Figure 8). Overall, only 5% of VIFs for RU were above the threshold of 10 (12 out of 240), however we found that around 20% of VIFs for TU and V/TU were above 10 (53 and 47 out of 240, respectively), and more than half of VIFs for V were above 10 (128 out of 240). Note that inferences are valid even for regressors with a high VIF since the estimate of the beta coefficients is still unbiased and thus the type I error rate is preserved [1]. However, the inflated variance of the beta estimates might reduce the power of the analysis. To investigate this possibility, we re-analyzed the data using four new GLMs that were nearly identical to GLM 1, with the only difference that each new GLM had a single parametric modulator at trial onset rather than four. Thus there was one GLM with RU, one with TU, one with V, and one with V/TU. As before, we thresholded single voxels at $p < 0.001$. Since we are not using these results for ROI selection, we report uncorrected whole-brain contrasts.

We found the same network of brain regions for RU and TU (Supplementary Figure 9A and B, respectively) and no regions for V/TU. For V, we found clusters in medial PFC (Supplementary Figure 9C), which is consistent with previous reports of value coding in this region [2, 3]. ROI analysis using an anatomically defined vmPFC region (as a conjunction of Superior frontal gyrus, medial orbital; Superior frontal gyrus, medial; and Gyrus rectus from the AAL2 atlas [4, 5]) showed a significant positive effect of V in left vmPFC ($t(30) = 2.14, p = 0.04$, t-test of ROI-averaged betas across subjects). Since the value-coding function of this region has been characterized extensively [6, 7] and since our primary interest was in the role of uncertainty in guiding exploration, we chose not to pursue this finding.

Reaction times and decision value

One potential confound of our decision value result (GLM 2) in motor cortex is reaction time (RT). When including RT's as a parametric modulator in addition to DV (GLM 2A), we found no effect of DV in motor cortex (no voxels

survived cluster FWE correction). Note, however, that the sequential sampling framework predicts a strong relationship between DV and RT's: when DV is close to zero, the two options are similar to each other and hence it takes longer for the evidence accumulator to reach a decision bound. This prediction was manifested in our data (coefficient $= -0.006, F(1,9717) = 23.8, p = 0.000001$, mixed effects linear regression: RT ~ 1 + DV + (1 + DV | SubjectID)), indicating that the negative result could be due to RT's capturing some of the shared variance in the BOLD signal.

To account for this possibility, we performed random effects Bayesian model comparison [8] between the GLM with DV alone (GLM 2), the GLM with both DV and RT (GLM 2A), and a GLM with RT alone (GLM 2B) in the left motor cortex ROI identified by GLM 2 (Figure 5A). Specifically, following our previous work [9], we approximated the log model evidence as -0.5 * BIC, where the BIC was computed based on the residual variance of the GLM fits within a 10 mm sphere around the peak voxel in left M1 from GLM 2. To prevent circularity [10], we performed this using leave-one-subject-out cross-validation: for each subject, we computed the BIC in the peak ROI from the group-level DV contrast computed using all other subjects. Since SPM fits each subject separately, this means that we used independent data for ROI selection and model comparison, resulting in an unbiased analysis. To ensure the validity of our inference, we confirmed that the resulting ROIs were highly overlapping (Supplementary Figure 10), with all but one subject having the same left M1 ROI as the contrast using all subjects (Figure 5A, MNI [-38 -8 62]). This analysis strongly favored GLM 2A (PXP = 0.96) over GLM 2 (PXP = 0) and GLM 2B (PXP = 0.04). This indicates that the BOLD signal in left M1 is best explained by combination of DV and RT, rather than RT or DV alone, pointing to decision value coding in motor cortex above and beyond RT's.

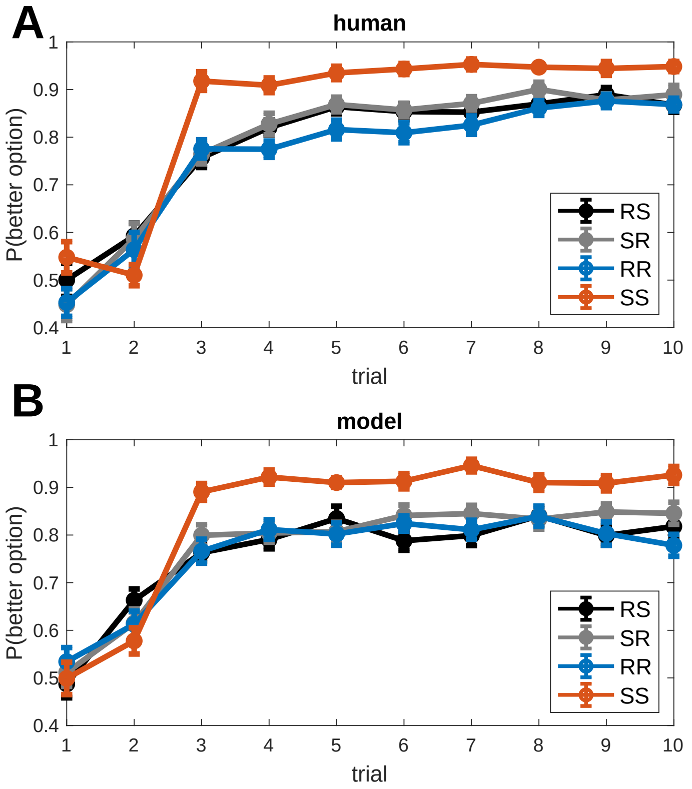

Supplementary Figure 1. **Learning curves** for human (A) and model (B) data. The better option is defined as the option with the greater expected reward $\mu(k)$. Error bars are cross-subject standard errors.

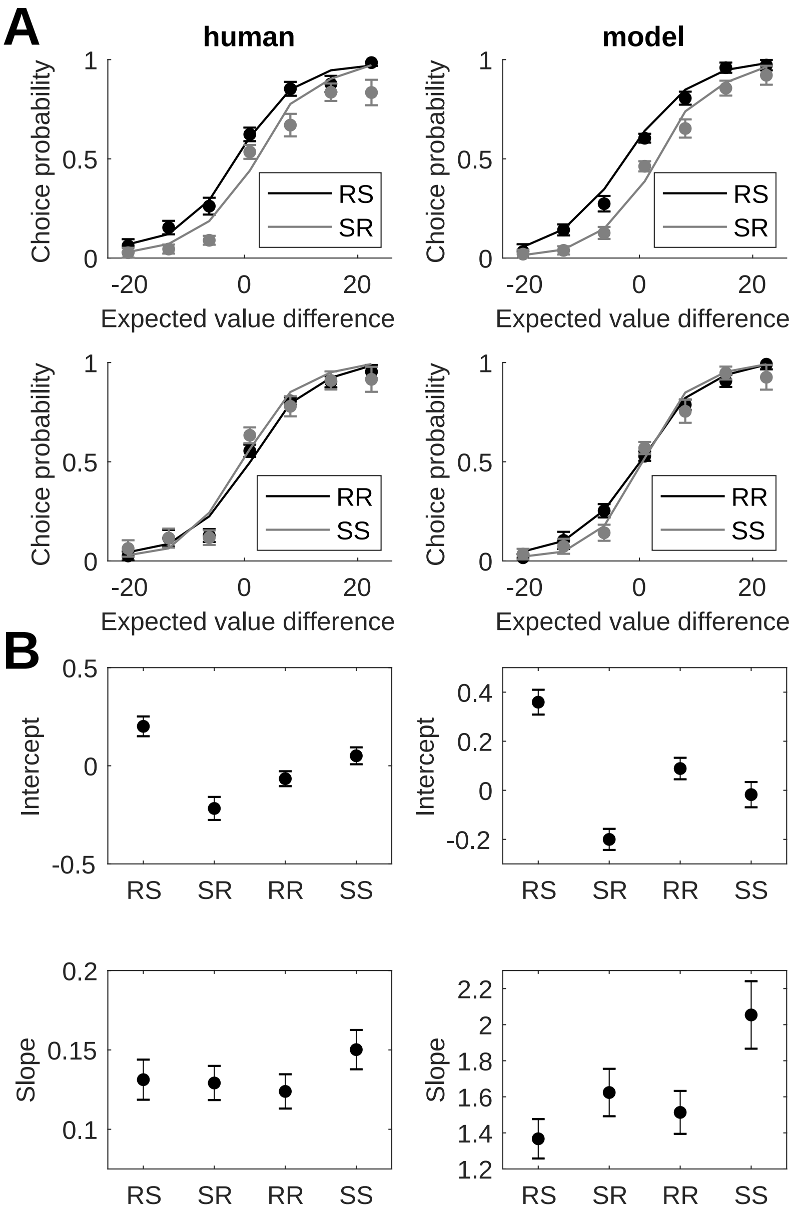

Supplementary Figure 2. **Choice probability functions (A) and probit regression results (B)** for human (left) and model (right) data. Error bars are cross-subject standard errors.

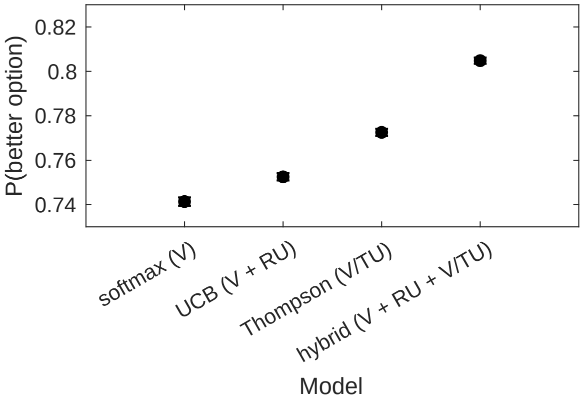

Supplementary Figure 3. **Performance comparison** of different exploration strategies. Simulation results from running different models generatively with subject-specific fitted coefficients. Error bars indicate s.e.m. across simulations.

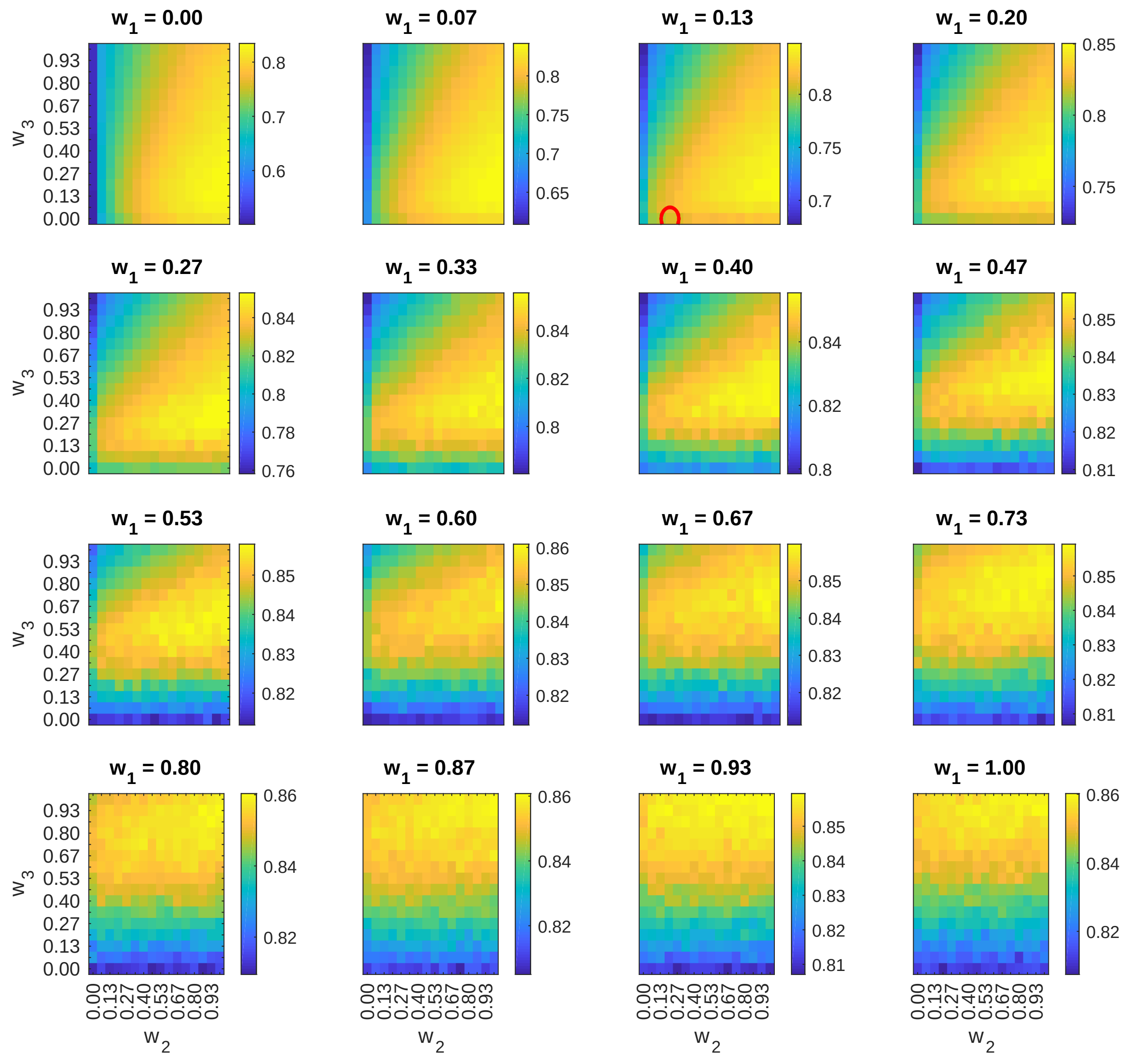

Supplementary Figure 4. **Performance comparison** of simulations of the UCB/Thompson hybrid model (Eq. 4) with different parameter settings **w**. Color scale indicates P(better option), averaged across simulations. Red circle denotes the fitted fixed effects coefficients.

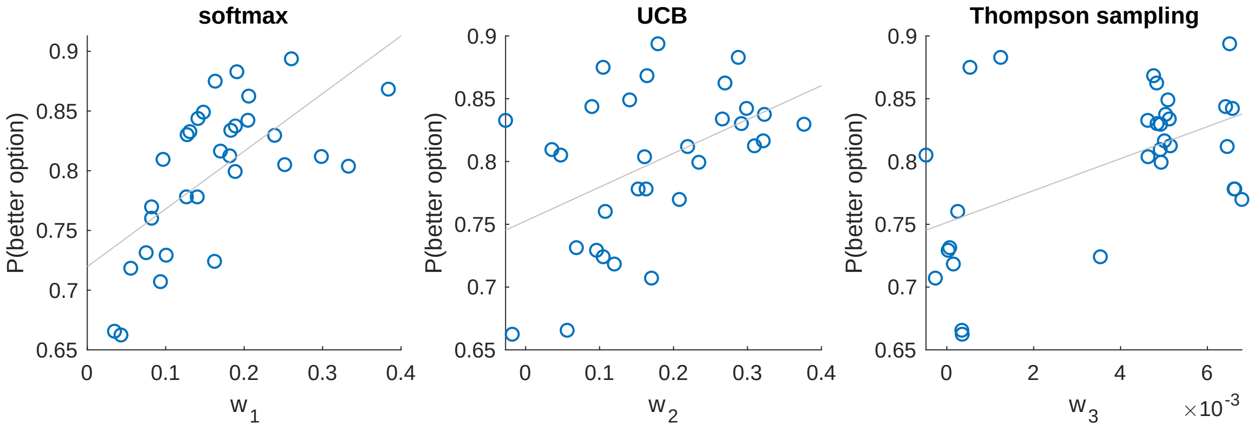

Supplementary Figure 5. **Subject performance based on exploration strategy**. Correlation between subject performance and fitted subject-specific coefficients (Eq. 4), indicating greater reliance on the corresponding strategy.

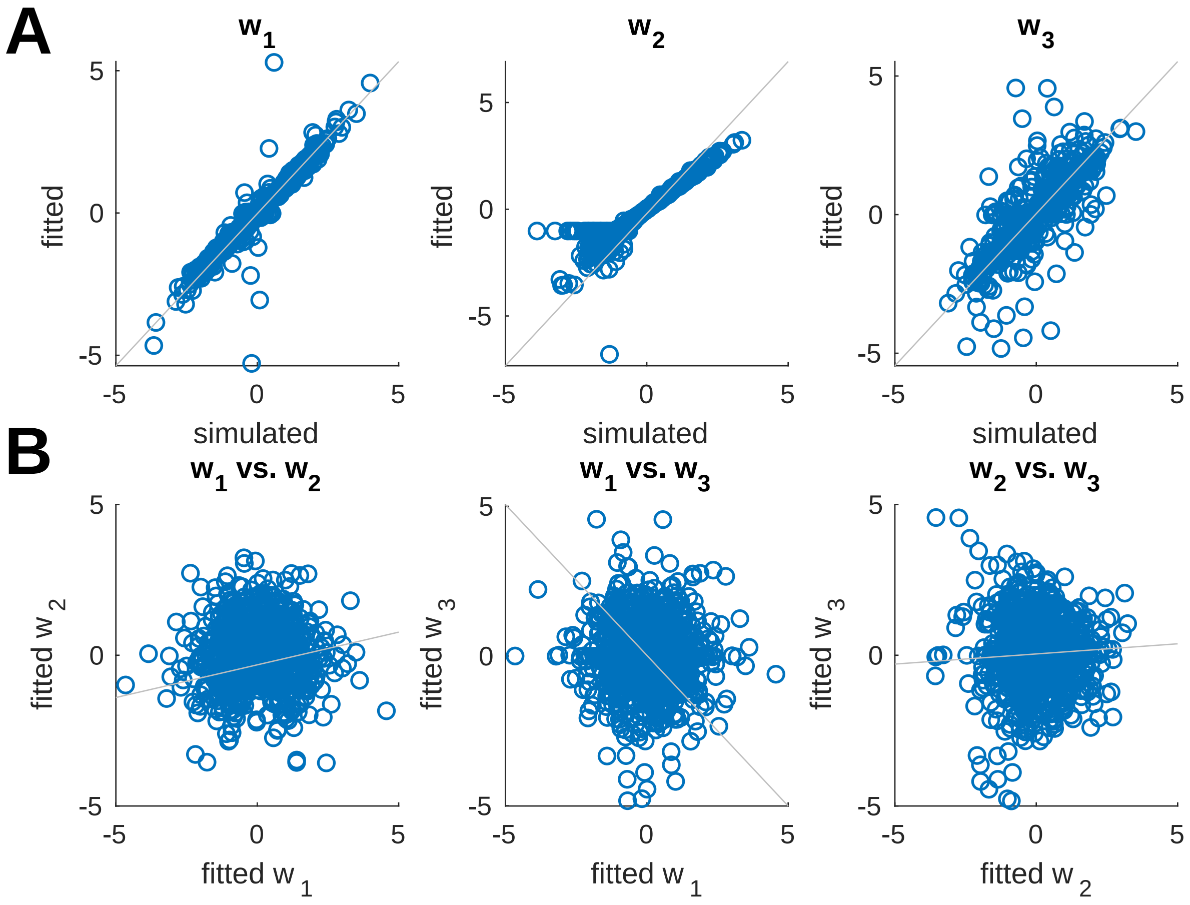

Supplementary Figure 6. **Parameter recoverability**.

(A) Correlation between simulated and fitted parameters.

(B) Correlation between fitted parameters.

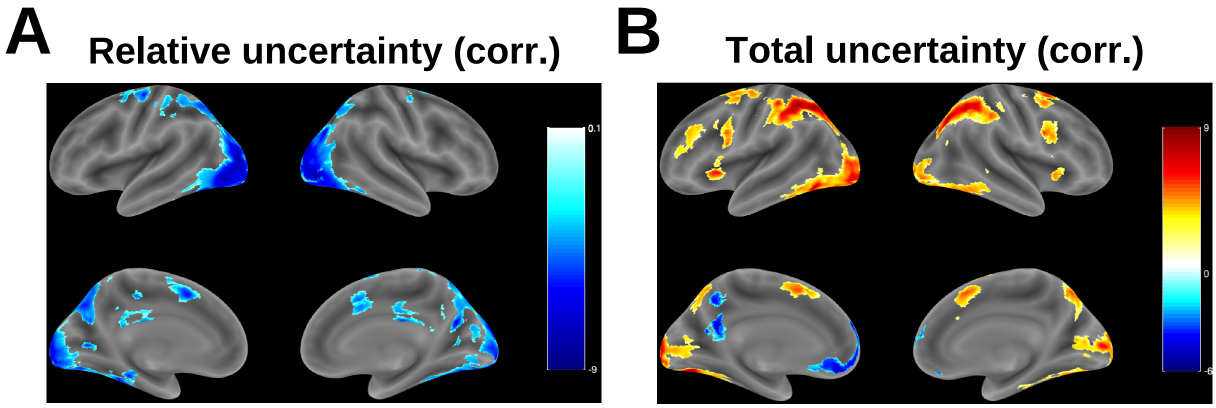

Supplementary Figure 7. **Corrected GLM 1 contrasts** with single voxels thresholded at $p < 0.001$ and cluster FWE correction applied at significance level $\alpha = 0.05$.

(A) Relative uncertainty ($|\mathrm{RU}_t|$) contrast. See Supplementary Table 3.

(B) Total uncertainty (TU_t) contrast. See Supplementary Table 4.

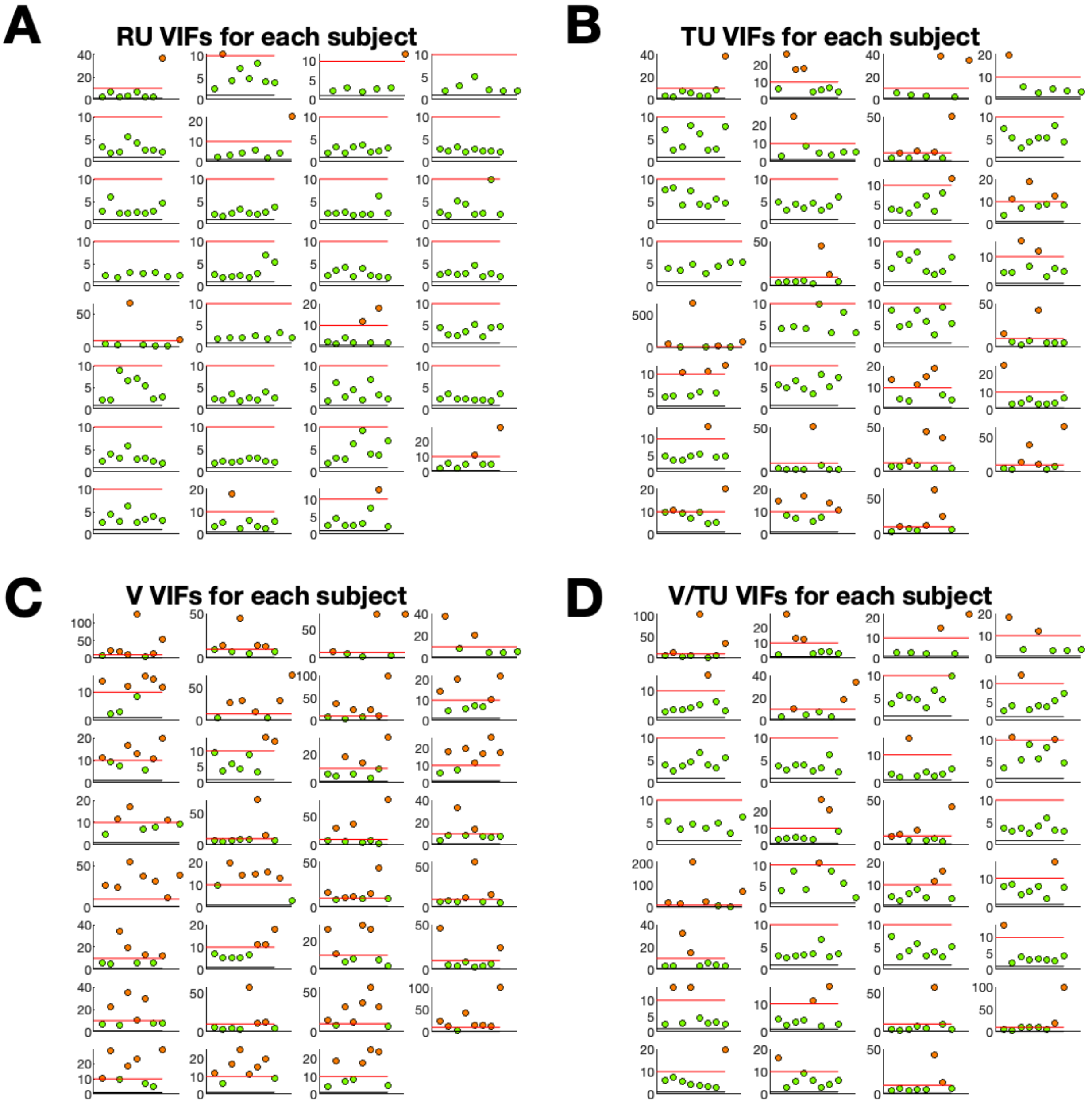

Supplementary Figure 8. **Variance inflation factors** (VIFs) for parametric modulators of the trial onset regressor in GLM 1. Each plot shows the VIFs for all runs of a given subject. Green circles correspond to runs with VIF <= 10, red circles correspond to runs with VIF > 10. A red horizontal line denotes the cutoff at 10.

(A) relative uncertainty (RU),

(B) total uncertainty (TU),

(C) value difference (V),

(D) value difference scaled by total uncertainty (V/TU).

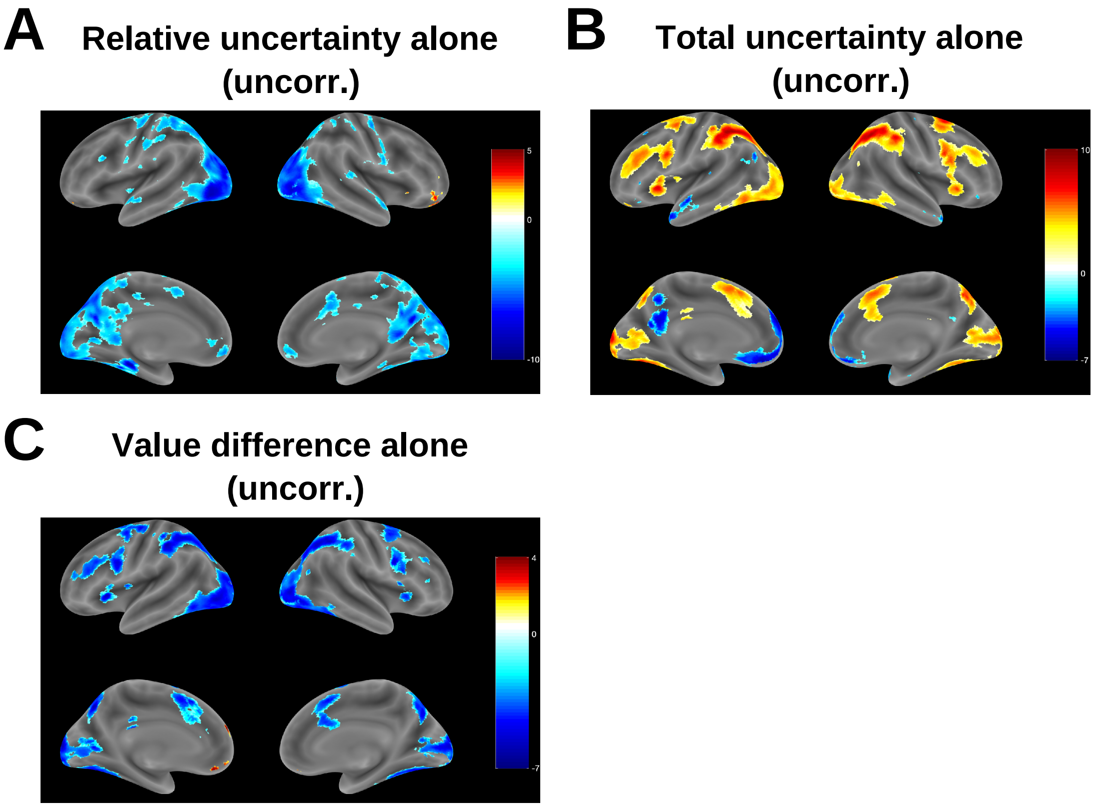

Supplementary Figure 9. **Contrasts from GLMs with a single parametric modulator.**

(A) Uncorrected whole-brain RU contrast when only RU was included as a parametric modulator. Compare with Figure 3A.

(B) Uncorrected whole-brain TU contrast when only TU was included as a parametric modulator. Compare with Figure 4A.

(C) Uncorrected whole-brain V contrast when only V was included as a parametric modulator.

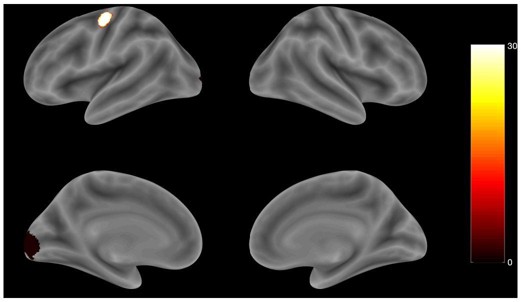

Supplementary Figure 10. **Heatmap of ROIs from leave-one-subject-out GLM 2 DV contrasts.**

Overlay of spherical ROIs around the peak voxel from the group-level DV contrast from GLM 2 using leave-one-subject-out cross-validation. Colorbar indicates how many folds each voxel was part of.

Model	Regressors	AIC	BIC	LL	Deviance
Softmax	V	8414.73	8400.37	-4198.18	8396.37
UCB	V + RU	7982.34	7953.62	-3972.81	7945.62
Thompson sampling	V/TU	8315.20	8300.84	-4148.42	8296.84
UCB/Thompson hybrid	V + RU + V/TU	6655.89	6612.80	-3300.40	6600.80

Supplementary Table 1. **Model comparison** between different exploration strategies, which can be thought of as lesioned versions of the UCB/Thompson hybrid model (Eq. 4). Lower AIC, BIC, and deviance indicate better fit. AIC = Akaike information criterion; BIC = Bayesian information criterion; LL = maximized log likelihood.

Regressor	Event	Duration	Pmods	Which trials
**GLM 1: RU, TU, V, V/TU**				
trial_onset	trial onset	0 s	$\lvert RU_t \rvert$, TU_t, $\lvert V_t \rvert$, $\lvert V_t \rvert / TU_t$	non-timeout
trial_onset_timeout	trial onset	0 s		timeout
trial_onset_chose_1	trial onset	0 s		chose arm 1
button_press	reaction time	0 s		all
feedback_onset	feedback onset	0 s		all
**GLM 2: DV**				
trial_onset	trial onset	0 s	$\lvert DV_t \rvert$	non-timeout
trial_onset_timeout	trial onset	0 s		timeout
trial_onset_chose_1	trial onset	0 s		chose arm 1
button_press	reaction time	0 s		all
feedback_onset	feedback onset	0 s		all

Supplementary Table 2. **GLM definitions**. GLMs used to analyze the fMRI data in the main text.

Sign	Brain region	BA	Extent	*t*-value	MNI coord.
Negative	Middle occipital gyrus (L)	18	27153	-9.164	-30 -92 6
	Cerebellum (L)	37	27153	-8.433	-46 -52 -30
	Inferior occipital gyrus (R)	19	27153	-8.398	40 -84 -8
	Precentral gyrus (L)	6	1066	-6.702	-38 -16 68
	Superior frontal gyrus, dorsolateral (L)	6	1066	-4.582	-24 -6 48
	Superior frontal gyrus, dorsolateral (L)	6	1066	-4.075	-20 -10 76
	Supplementary motor area (L)	32	860	-6.231	-10 8 46
	Middle cingulate & paracingulate gyri (R)	32	860	-5.291	10 14 38
	Posterior cingulate gyrus (R)		530	-5.550	4 -22 28
	Left cerebral white matter (L)		530	-5.380	-16 -28 38
	Posterior cingulate gyrus (L)	26	530	-4.700	-6 -42 24
	Middle frontal gyrus (R)		732	-5.197	38 -4 68
	Supplementary motor area (R)	6	732	-5.169	16 2 62

Supplementary Table 3. **GLM 1 results: relative uncertainty**. Brain regions in which the BOLD signal tracks $|\text{RU}_t|$ from GLM 1 (corresponding to Supplementary Figure 7A). Anatomical labels and MNI coordinates are based on peak voxels (maximum *t*-statistic), with up to three peaks extracted per cluster (minimum separation of 20 voxels). Single voxels were thresholded at $p < 0.001$ and whole-brain cluster FWE correction was applied at significance level $\alpha = 0.05$. Regions were labeled using the Automated Anatomical Labeling (AAL2) atlas, the SPM Anatomy Toolbox, and the CMA Harvard-Oxford atlas. MNI = Montreal Neurological Institute; BA = Brodmann area.

Sign	Brain region	BA	Extent	*t*-value	MNI coord.
Positive	Inferior parietal gyrus, excluding supramarginal and angular gyri (L)	40	25346	9.793	-42 -40 52
	Inferior parietal gyrus, excluding supramarginal and angular gyri (R)	40	25346	9.362	38 -42 44
	Middle occipital gyrus (R)	19	25346	9.111	32 -66 36
	Precentral gyrus (L)	44	785	7.353	-44 4 30
	Insula (L)	48	614	7.054	-28 20 2
	Precentral gyrus (R)	44	709	7.018	48 8 30
	Thalamus (L)		294	6.099	-4 -16 14
	Insula (R)	47	307	5.369	36 18 0
	Middle frontal gyrus (L)	46	704	5.152	-42 36 36
	Middle frontal gyrus (L)	46	704	4.405	-44 48 14
Negative	Superior frontal gyrus, dorsolateral (L)	10	1650	-6.957	-4 62 30
	Superior frontal gyrus, medial orbital (L)	11	1650	-6.448	-2 58 -8
	Gyrus rectus (L)	11	1650	-5.884	-4 36 -16
	Precuneus (L)	30	436	-5.904	-10 -54 16
	Precuneus (L)	30	436	-5.196	-10 -50 38

Supplementary Table 4. **GLM 1 results: total uncertainty**. Brain regions in which the BOLD signal tracks TU_t (corresponding to Supplementary Figure 7B). Notation and procedures as in Supplementary Table 3.

Sign	Brain region	BA	Extent	*t*-value	MNI coord.
Negative	Precentral gyrus (L)	6	721	-6.851	-38 -8 62
	Postcentral gyrus (L)	2	721	-4.303	-48 -38 56
	Superior frontal gyrus (L)		721	-4.014	-14 6 74

Supplementary Table 5. **GLM 2 results: decision value**. Brain regions in which the BOLD signal tracks $|DV_t|$ (corresponding to Figure 5A). Notation and procedures as in Supplementary Table 3.

Supplementary References

[1] Jeanette Mumford, Jean-Baptiste Poline, and Russell Poldrack. “Orthogonalization of Regressors in fMRI Models”. In: *PLoS ONE* 10 (Apr. 2015), e0126255.

[2] William H Alexander and Joshua W Brown. “Medial prefrontal cortex as an action-outcome predictor”. In: *Nature neuroscience* 14.10 (2011), p. 1338.

[3] Fabian Grabenhorst and Edmund T Rolls. “Value, pleasure and choice in the ventral prefrontal cortex”. In: *Trends in cognitive sciences* 15.2 (2011), pp. 56–67.

[4] N. Tzourio-Mazoyer et al. “Automated Anatomical Labeling of Activations in SPM Using a Macroscopic Anatomical Parcellation of the MNI MRI Single-Subject Brain”. In: *NeuroImage* 15.1 (2002), pp. 273–289. ISSN: 1053-8119. DOI: http://dx.doi.org/10.1006/nimg.2001.0978. URL: http://www.sciencedirect.com/science/article/pii/S1053811901909784.

[5] Edmund T. Rolls, Marc Joliot, and Nathalie Tzourio-Mazoyer. “Implementation of a new parcellation of the orbitofrontal cortex in the automated anatomical labeling atlas”. In: *NeuroImage* 122 (2015), pp. 1–5. ISSN: 1053-8119. DOI: http://dx.doi.org/10.1016/j.neuroimage.2015.07.075. URL: http://www.sciencedirect.com/science/article/pii/S1053811915006953.

[6] Nathaniel D Daw et al. “Cortical substrates for exploratory decisions in humans”. In: *Nature* 441.7095 (2006), p. 876.

[7] Seung-Lark Lim, John P O’Doherty, and Antonio Rangel. “The decision value computations in the vmPFC and striatum use a relative value code that is guided by visual attention”. In: *Journal of Neuroscience* 31.37 (2011), pp. 13214–13223.

[8] Lionel Rigoux et al. “Bayesian model selection for group studies-revisited”. In: *Neuroimage* 84 (2014), pp. 971–985.

[9] Momchil S Tomov, Hayley M Dorfman, and Samuel J Gershman. “Neural computations underlying causal structure learning”. In: *Journal of Neuroscience* 38.32 (2018), pp. 7143–7157.

[10] Nikolaus Kriegeskorte et al. “Circular analysis in systems neuroscience: the dangers of double dipping”. In: *Nature neuroscience* 12.5 (2009), p. 535.
