# Supplementary material for: Dissociable neural correlates of uncertainty underlie different exploration strategies
Source: Nat Commun. 2020 May 12;11:2371. doi: 10.1038/s41467-020-15766-z (PMC7217879; doi:10.1038/s41467-020-15766-z)
Supplement: Supplementary file 3 — Reporting Summary [file 41467_2020_15766_MOESM3_ESM.pdf]

nature research

Corresponding author(s): Momchil Tomov
Last updated by author(s): 11/04/2019

Reporting Summary

Nature Research wishes to improve the reproducibility of the work that we publish. This form provides structure for consistency and transparency in reporting. For further information on Nature Research policies, see Authors & Referees and the Editorial Policy Checklist.

Statistics

For all statistical analyses, confirm that the following items are present in the figure legend, table legend, main text, or Methods section.

n/a	Confirmed	
☐	☒	The exact sample size (*n*) for each experimental group/condition, given as a discrete number and unit of measurement
☐	☒	A statement on whether measurements were taken from distinct samples or whether the same sample was measured repeatedly
☐	☒	The statistical test(s) used AND whether they are one- or two-sided<br>*Only common tests should be described solely by name; describe more complex techniques in the Methods section.*
☐	☒	A description of all covariates tested
☐	☒	A description of any assumptions or corrections, such as tests of normality and adjustment for multiple comparisons
☐	☒	A full description of the statistical parameters including central tendency (e.g. means) or other basic estimates (e.g. regression coefficient) AND variation (e.g. standard deviation) or associated estimates of uncertainty (e.g. confidence intervals)
☐	☒	For null hypothesis testing, the test statistic (e.g. *F*, *t*, *r*) with confidence intervals, effect sizes, degrees of freedom and *P* value noted<br>*Give P values as exact values whenever suitable.*
☐	☒	For Bayesian analysis, information on the choice of priors and Markov chain Monte Carlo settings
☒	☐	For hierarchical and complex designs, identification of the appropriate level for tests and full reporting of outcomes
☐	☒	Estimates of effect sizes (e.g. Cohen's *d*, Pearson's *r*), indicating how they were calculated

Our web collection on statistics for biologists contains articles on many of the points above.

Software and code

Policy information about availability of computer code

Data collection	PsychoPy3
Data analysis	MATLAB, SPM12, CCNL fMRI pipeline (https://github.com/sjgershm/ccnl-fmri), custom scripts (https://github.com/tomov/Exploration-fMRI-Task)

For manuscripts utilizing custom algorithms or software that are central to the research but not yet described in published literature, software must be made available to editors/reviewers. We strongly encourage code deposition in a community repository (e.g. GitHub). See the Nature Research guidelines for submitting code & software for further information.

Data

Policy information about availability of data

All manuscripts must include a data availability statement. This statement should provide the following information, where applicable:

- Accession codes, unique identifiers, or web links for publicly available datasets
- A list of figures that have associated raw data
- A description of any restrictions on data availability

All behavioral data and analysis code are available https://github.com/tomov/Exploration-fMRI-Task. The raw fMRI data is available upon request.

Field-specific reporting

Please select the one below that is the best fit for your research. If you are not sure, read the appropriate sections before making your selection.

☐ Life sciences ☒ Behavioural & social sciences ☐ Ecological, evolutionary & environmental sciences

Behavioural & social sciences study design

All studies must disclose on these points even when the disclosure is negative.

Study description	Human subjects performed a two-armed banded task while undergoing fMRI scanning.
Research sample	We used a convenience sample consisting of Harvard undergraduates and members of the Cambridge community.
Sampling strategy	we use a sample size consistent with current practices in the field
Data collection	Scanning was carried out on a 3T Siemens Magnetom PrismaMRI scanner with the vendor 32-channel head coil (Siemens Healthcare, Erlangen, Germany) at the Harvard University Center for Brain Science Neuroimaging. Behavioral data was collected on a MacBook air connected to the scanner button box.
Timing	All data was collected in July and August 2018
Data exclusions	We excluded seven scanner runs due to excessive motion and one run during which the subject fell sleep
Non-participation	no participants dropped out
Randomization	all participants were assigned to the same conditions, which were randomized within participant

Reporting for specific materials, systems and methods

We require information from authors about some types of materials, experimental systems and methods used in many studies. Here, indicate whether each material, system or method listed is relevant to your study. If you are not sure if a list item applies to your research, read the appropriate section before selecting a response.

Materials & experimental systems

n/a	Involved in the study	
☒	☐	Antibodies
☒	☐	Eukaryotic cell lines
☒	☐	Palaeontology
☒	☐	Animals and other organisms
☐	☒	Human research participants
☒	☐	Clinical data

Methods

n/a	Involved in the study	
☒	☐	ChIP-seq
☒	☐	Flow cytometry
☐	☒	MRI-based neuroimaging

Human research participants

Policy information about studies involving human research participants

Population characteristics	We recruited 31 subjects (17 female) fromthe Cambridge community. All subjects were healthy, ages 18-35, right-handed, with normal or corrected vision, and no neuropsychiatric pre-conditions
Recruitment	Participants were recruited using posters around campus and email listserves
Ethics oversight	All subjects received written consent and the study was approved by the Harvard Institutional Review Board.

Note that full information on the approval of the study protocol must also be provided in the manuscript.

Magnetic resonance imaging

Experimental design

Design type	event related
Design specifications	Eight runs per participant, four blocks per run, 10 trials per block. Intervals between blocks were six seconds. Each run was about eight minutes
Behavioral performance measures	Button press, button press times. We ensured all subjects achieved above chance performance

Acquisition

Imaging type(s) | Functional

Field strength | 3T

Sequence & imaging parameters | A T1-weighted high-resolution multi-echo magnetization-prepared rapid-acquisition gradient echo (ME-MPRAGE) anatomical scan (van der Kouwe et al., 2008) of the whole brain was acquired for each subject prior to any functional scanning (176 sagittal slices, voxel size = 1.0 x 1.0 x 1.0 mm, TR = 2530 ms, TE = 1.69 - 7.27 ms, TI = 1100 ms, flip angle = 7∘, FOV = 256 mm). Functional images were acquired using a T2*-weighted echo-planar imaging (EPI) pulse sequence that employed multiband RF pulses and Simultaneous Multi-Slice (SMS) acquisition (Moeller et al., 2010; Feinberg et al., 2010; Xu et al., 2013). In total, 8 functional runs were collected for each subject, with each run corresponding to 4 task blocks, one in each condition (84 interleaved axial-oblique slices per whole brain volume, voxel size = 1.5 x 1.5 x 1.5 mm, TR = 2000 ms, TE = 30 ms, flip angle = 80∘, in-plane acceleration (GRAPPA) factor = 2, multi-band acceleration factor = 3, FOV = 204 mm). The initial 5 TRs (10 s) were discarded as the scanner stabilized. Functional slices were oriented to a 25 degree tilt towards coronal from AC-PC alignment. The SMS-EPI acquisitions used the CMRR-MB pulse sequence from the University of Minnesota.

Area of acquisition | Whole brain

Diffusion MRI ☐ Used ☒ Not used

Preprocessing

Preprocessing software | Functional images were preprocessed and analyzed using SPM12 (Wellcome Department of Imaging Neuroscience, London, UK). Each functional scan was realigned to correct for small movements between scans, producing an aligned set of images and a mean image for each subject.

Normalization | The high-resolution T1-weighted ME-MPRAGE images were then co-registered to the mean realigned images and the gray matter was segmented out and normalized to the gray matter of a standard Montreal Neurological Institute (MNI) reference brain. The functional images were then normalized to the MNI template (resampled voxel size 2 mm isotropic)

Normalization template | MNI

Noise and artifact removal | The functional images were patially smoothed with a 8 mm full-width at half-maximum (FWHM) Gaussian kernel, high-pass filtered at 1/128 Hz, and corrected for temporal autocorrelations using a first-order autoregressive model

Volume censoring | not applicable

Statistical modeling & inference

Model type and settings | mass univariate GLM, random effects

Effect(s) tested | we used a factorial design, where each arm was either risky or safe. In our model free analysis, we tested the effect of block condition. In our model-based analysis, we tested the effect of different uncertainty estimates derived from the model.

Specify type of analysis: ☐ Whole brain ☐ ROI-based ☒ Both

Anatomical location(s) | Based on prior studies

Statistic type for inference (See Eklund et al. 2016) | We used cluster FWE correction with p = 0.001 and alpha = 0.05 and report the voxel-wise t-statistics. We also report uncorrected contrast

Correction | We used cluster FWE correction, as well as averaging within ROI for our analysis using prior ROIs

Models & analysis

n/a	Involved in the study	
☒	☐	Functional and/or effective connectivity
☒	☐	Graph analysis
☒	☐	Multivariate modeling or predictive analysis
